# Obesity and Its Role in Fetal Programming—A Narrative Review

**DOI:** 10.3390/nu17233704

**Published:** 2025-11-26

**Authors:** Radzisław Mierzyński, Elżbieta Poniedziałek-Czajkowska, Kamila Świda, Katarzyna Mierzyńska

**Affiliations:** 1 Department of Obstetrics and Perinatology, Medical University of Lublin, 20-059 Lublin, Poland; 2 Medical University of Lublin, 20-059 Lublin, Poland

**Keywords:** obesity, fetal programming, pregnancy, nutritional status, metabolic diseases, cardiovascular diseases

## Abstract

The prevalence of maternal obesity is rapidly increasing, which represents a major public health concern worldwide. Currently more than 50% of all adult women are overweight or obese, and this trend is reflected in women of child-bearing age. Maternal obesity is characterized by metabolic dysfunction and chronic inflammation, and is associated with health problems in both the mother and the offspring. Intrauterine programming occurs during embryonic and fetal development, a critical period not only for the formation of tissues and organs but also for the etiology of diseases later in life. The principal mechanisms underlying fetal programming in the offspring of obese mothers appear to involve DNA methylation and chromatin remodeling within progenitor cells. Aberrant DNA methylation patterns have been identified in genes involved in insulin signaling, lipid metabolism, and appetite regulation in the placenta and fetal tissues. Histone modifications, such as acetylation and methylation of histone tails, may also play a crucial role in modulating chromatin structure and accessibility of transcriptional machinery to DNA. The persistence of such modifications throughout life, and potentially across generations, can lead to permanent alterations in gene expression, thereby contributing to the intergenerational transmission of metabolic disorders. The aim of this paper is to present an overview of the current knowledge regarding the effects of maternal obesity on fetal development and the occurrence of fetal complications, as well as long-term complications observed in adulthood related to intrauterine exposure to maternal obesity, including hypertension and cardiovascular diseases, impaired insulin secretion and resistance, diabetes mellitus, and metabolic syndrome. The mechanisms underlying fetal programming are also discussed.

## 1. Introduction

In recent decades, a dramatic increase in the incidence of overweight and obesity among women of reproductive age has been observed. It has been noticed that more than 50% of all adult women are overweight or obese, and similar patterns are observed among women of reproductive age [1]. The pooled prevalence of combined overweight and obesity during pregnancy was 43.8% (95% CI: 42.2–45.4%), representing nearly half of all pregnancies. A rising trend, consistent with the global pattern, is observed all over the world. North America exhibited the highest prevalence, with obesity at 18.7% (95% CI: 15.0–23.2%) and combined overweight/obesity at 47.0% (95% CI: 45.7–48.3%), whereas Asia showed the lowest prevalence, with obesity at 10.8% (95% CI: 7.0–16.5%) and combined overweight/obesity at 28.5% (95% CI: 18.3–41.5%). The prevalence of maternal obesity and combined overweight/obesity increased annually by 0.34% and 0.64%, respectively (*p* < 0.001) [1]. The widespread availability of highly processed foods, combined with reduced physical activity, represents a major public health concern. This imbalance leads to impaired energy homeostasis and contributes to the growing epidemic of obesity [1].

The overweight and obesity usually are defined using the body mass index (BMI), with values ≥ 25 kg/m^2^ identifying overweight and ≥30 kg/m^2^ defining obesity. In pregnancy, excess maternal weight, regardless of pre-existing chronic disease, are the risk factors of numerous obstetric and neonatal complications: gestational hypertension and preeclampsia, gestational diabetes mellitus (GDM), caesarean section, and labour induction. Similarly, neonatal risks rise up with the maternal BMI augmentation including preterm birth below 32 weeks of gestation, macrosomia, transient tachypnea, sepsis, and an intensive care unit admission [2,3]

Fetal programming refers to the process by which environmental conditions during critical periods of intrauterine development induce long-lasting structural, metabolic, and functional adaptations in the developing fetus. Fetal programming is a central concept within the Developmental Origins of Health and Disease (DOHaD) framework and is implicated in the pathogenesis of diseases observed in adulthood [4].

During the first months of pregnancy, the fetus undergoes a process of rapid differentiation. The numerous cell divisions occurring during this period of fetal life, known as the critical developmental window, result in a particularly high sensitivity of cells to external factors [3]. It can be proposed that prenatal metabolic activity and fetal growth are adapted to anticipated postnatal metabolic conditions, which are largely determined by intrauterine nutrient availability. When considering nutrient supply, the regulatory role of placental transport must also be emphasized, as it provides key insights into the mechanisms responsible for abnormal fetal development and metabolic programming. Placental nutrient sensing regulates trophoblast growth as well as the transfer of nutrients, oxygen, hormones, and metabolic signals, thereby shaping the intrauterine environment to which the fetus adapts. Alterations in placental transport capacity, particularly of glucose, lipids, amino acids, and fatty acids, have been implicated in fetal overnutrition and in the long-term propensity for obesity and metabolic disease [5].

A well-balanced diet during pregnancy, particularly in the first trimester, is crucial for establishing a healthier epigenetic pattern and reducing the risk of both pregnancy complications and metabolic disorders in the offspring. Maternal overweight and obesity are significant factors contributing to the etiology of chronic diseases in offspring, although the exact mechanisms have not yet been fully elucidated. Newborns and infants born to overweight mothers typically exhibit higher body fat content. Enhanced adipogenesis during fetal development predisposes these offspring to obesity in childhood, metabolic syndrome, and cardiovascular diseases in adulthood [6].

Epidemiological and clinical studies have also described that neonates exposed to maternal overnutrition and/or obesity during breastfeeding also have an increased risk of metabolic syndrome-related diseases later in the future [7]. This phenomenon is referred to as “fetal programming” or “developmental origin of adult disease”.

Most of the published studies on this topic focus on selected, individual fetal complications associated with fetal programming that may manifest later in the lives of children born to obese mothers, discussing these issues in detail. However, there is a lack of publications that address this problem more broadly. A comprehensive review and discussion, within a single article, of the mechanisms linking fetal programming to the occurrence of multiple complications in the offspring of obese mothers provides a wider perspective on these issues and supports further research on fetal programming mechanisms and potential interventions.

The aim of this paper is to present an overview and to systemize of the current knowledge of the effects of obesity on fetal development and the occurrence of fetal complications, as well as complications observed in adulthood, related to intrauterine exposure to maternal obesity: hypertension and cardiovascular diseases, insulin secretion and resistance, diabetes mellitus, and metabolic syndrome. The mechanisms underlying fetal programming are also discussed.

### Methods

This narrative review involved a comprehensive search of several major databases, including PubMed, Medline and ScienceDirect. The databases were searched from the first records until June 2025 using MeSH Terms alone or in combination such as: “obesity”, “fetal programming”, “nutritional status”, “pregnancy”, “metabolic diseases” as keywords. All identified publications underwent a detailed and critical assessment, with intentional emphasis placed on studies most relevant to the primary topic. Duplicate records, conference abstracts, and editorial letters were removed from consideration. Subsequently, full-text clinical investigations, review papers, and meta-analyses were systematically reviewed and appraised. The references included in the selected publications were also analyzed to find additional relevant publications. Only articles in English were considered.

## 2. Fetal Programming

Fetal programming occurs when the optimal intrauterine environment for the developing fetus is disrupted by external factors, particularly during critical periods of organ development. Epigenetic patterns established in response to intrauterine environmental conditions are transmitted during successive cell divisions in the developing organism, thereby becoming stabilized and limiting the potential for further modification.

Although the precise mechanisms of this process remain not fully understood, the detrimental effects of intrauterine stress have been implicated in the etiology of various diseases in the offspring, including atopic disorders, cardiovascular diseases, and malignancies such as lymphomas, liver cancer, and testicular cancer [4,8].

Factors initiating fetal programming include unhealthy maternal behaviors, such as tobacco smoking, lack of physical activity, and psychosocial stress; as well as maternal diseases, including endocrine disorders, preeclampsia, neurological disorders, depression, infections, and nitrosative or oxidative stress [9,10]. The epigenetic marks established under the influence of the intrauterine environment are propagated through cell divisions during development, leading to their stabilization and restricting the potential for subsequent reprogramming.

The term “*fetal programming*” was introduced by the British epidemiologist David Barker, who investigated the relationship between low birth weight and an increased risk of ischemic heart disease in adult life [11]. It is the general idea, which holds that environmental influences during embryonic and fetal development can modify key physiological processes or endocrine disorders can influence cellular growth patterns, hormonal signaling, and gene expression. The most important thing is that the resetting can continue into adulthood and even affect the next generation, creating a transgenerational non-genetic disorder [12]. A well-known example of this phenomenon comes from the Dutch Hunger Winter of 1944–1945, during which individuals exposed to famine early in gestation were later found to have a markedly increased likelihood of adult obesity [13].

The mechanisms responsible for fetal programming remain incompletely understood. It has been suggested that the primary targets of fetal programming are adipocytes and pancreatic β-cells. Several publications have demonstrated that modulation of epigenetic mechanisms can modify cell fate and identity, as well as the expression of cell type-specific genes during the formation of both adipocytes and β-cells [14]. Adipogenesis and β-cell neogenesis in rodents occur predominantly in the second half of pregnancy, accelerate in the early postnatal period, and remain active after weaning. The adverse effects of maternal obesity appear to influence developmental stages when precursor cells exhibit high plasticity, that is, the ability to adapt to changes in their microenvironment, and when epigenetic remodeling is particularly dynamic and sensitive to nutritional and hormonal signals [15].

The main mechanisms implicated in fetal programming are epigenetic modifications, altered placental function, dysregulated fetal endocrine and metabolic signaling, and inflammatory responses.

### 2.1. Epigenetic Factors

Epigenetic factors are believed to play a crucial role in this process. The concept of “epigenetics” was first described by the British biologist Conrad Hal Waddington, who, in 1942, used the term to describe the phenotype as the result of interactions between genes and the environment [16].

Epigenetic modifications represent a pivotal mechanism through which the intrauterine environment, influenced by maternal obesity and nutritional status, exerts long-term effects on fetal gene expression without modifying the DNA sequence itself. The most extensively studied epigenetic changes mechanisms include DNA methylation, histone phosphorylation, acetylation, and methylation; as well as microRNA (micro-ribonucleic acid)—mediated translational inhibition [17,18]. These modifications are crucial for maintaining genomic stability and serve as key regulatory mechanisms of cellular differentiation during embryogenesis and fetal development [19].

DNA methylation is a key epigenetic mechanism implicated in fetal programming, modulating gene expression in response to the in utero environment. It is a post-replicative modification of DNA that primarily contributes to gene silencing. It results from the transfer of a methyl group, by DNA methyltransferase (DNMTs), to cytosine residues, mainly within CpG islands, forming 5-methylcytosine (5mC) [20]. Another important modification of cytosine is 5-hydroxymethylcytosine (5hmC), generated through the catalytic activity of the ten-eleven translocation (TET) family of methylcytosine dioxygenases. This molecule acts as an intermediate in the DNA demethylation process and is predominantly localized in in active transcriptional regulatory regions [21]. The pattern of DNA methylation is established during the early stages of embryonic development and is maintained throughout the lifetime by DNA methyltransferases. This methylation pattern is tissue-specific and heritable [22]. In obese patients, aberrant DNA methylation patterns have been observed in genes regulating insulin signaling (insulin receptor substrate-1- IRS1, insulin-like growth factor 2-IGF2), lipid metabolism (peroxisome proliferator-activated receptor gamma—PPARγ, fatty acid synthase—*FASN*), and appetite control (leptin receptor—LEP, melanocortin 4 receptor—MC4R) in the placenta and fetal tissues. In human studies, maternal pre-pregnancy BMI has been associated with differential CpG methylation in cord blood that partially mediates the association between maternal adiposity and obesity in offspring [23]. In the placenta, specific methylation loci correlate with birthweight and expression of developmental and metabolic genes, indicating that epigenetic modifications at the maternal–fetal interface contribute to fetal growth programming [24]. In animal models, maternal high-fat diet (HFD) induces hypermethylation of the insulin receptor substrate 2 (Irs2) gene and hypomethylation of hypomethylated mitogen-activated protein kinase 4 (Map2k4) gene in the offspring liver, which is associated with reduced insulin receptor substrate expression and increased glucose intolerance [25].

Moreover, maternal obesity has been described to upregulate DNA methyltransferases (e.g., DNMT3A) in oocytes, leading to global hypermethylation that may persist into subsequent generations. Longitudinal studies of infants born to obese mothers reveal methylation changes maintained across the first year of life in genes related to fatty acid transport, mitochondrial bioenergetics, and developmental processes [26]. Thus, altered DNA methylation can predispose offspring to insulin resistance, adiposity, and metabolic dysregulation in future life [23].

Histone modifications, such as acetylation and methylation of histone tails, also play a crucial role in modulating chromatin structure and accessibility of transcriptional machinery to DNA. Animal studies in non-human primates have shown that a maternal high-fat diet induces hyperacetylation of fetal hepatic histone H3 (e.g., at H3K14), suggesting that histone acetylation marks may respond dynamically to in utero nutritional cues [27]. Moreover, in the context of maternal overnutrition, imbalances in histone acetyltransferase (HAT) and histone deacetylase (HDAC) expression in fetal tissues such as liver and placenta have been documented, indicating that dysregulation of the acetylation/deacetylation switch may contribute to metabolic programming [28]. These modifications of histone marks influence the transcriptional landscape of genes involved in energy metabolism, cell differentiation, and organ development, thereby establishing a molecular basis for long-term phenotypic modifications in the offspring. Altered maternal nutrient supply, including excessive intake of saturated fats or deficiencies in methyl donors (e.g., folate, choline), can affect histone-modifying enzymes like histone acetyltransferases and deacetylases, thereby influencing gene expression patterns critical for organ development and metabolic function [29].

Furthermore, maternal obesity has been correlated with modifications in the expression of non-coding RNAs, particularly microRNAs (miRNAs), which post-transcriptionally regulate mRNA stability and translation. Sanli et al. reported dysregulation of miR-122 and miR-33, involved in hepatic lipid metabolism, in the offspring of obese dams in both human and animal studies [17].

These epigenetic marks, established during fetal development, often remain stable throughout life and may even be transmitted across generations, thereby contributing to a cycle of metabolic disease risk [29].

### 2.2. Altered Placental Function

It is proposed, that placental dysfunction acts as a mediator of fetal programming, linking maternal metabolic state to altered fetal growth, and long-term health. Pregnancy complications modify the activity of placental transporters and enzymes, as well as disrupt hormonal secretion [10]. These disturbances lead to a reduction in the supply of essential substances to the fetus, ultimately impairing normal fetal development and initiating epigenetic modifications. In pregnancies complicated by maternal obesity or a high-fat diet, placental development and function are frequently impaired [30].

Morphological changes such as villous immaturity, vascular malperfusion, and increased placental weight have been described. Functionally, there is evidence of dysregulated expression of nutrient transporters, including glucose transporters (glucose transporter type 1 and 3-GLUT1, GLUT3), amino acid transporters (sodium-coupled neutral amino acid transporter—SNAT, large amino acid transporter—LAT), and fatty acid transporters (fatty acid transporters—FAT/CD36). These alterations may result in excessive or unbalanced nutrient delivery to the fetus, promoting adipogenesis and abnormal fetal growth [31,32].

Pregnancies in obese patients are also associated with placental inflammation, characterized by elevated levels of pro-inflammatory cytokines (Interleukin-6, TNF-α) and activation of macrophages within placental tissue. These inflammatory processes can influence trophoblast invasion, angiogenesis, and hormone synthesis, disrupting the endocrine and immune functions of the placenta [32].

Moreover, maternal obesity affects placental mitochondrial function, leading to increased production of reactive oxygen species (ROS) and oxidative stress, which can further damage placental tissues and affect fetal development [30].

### 2.3. Dysregulated Fetal Endocrine and Metabolic Signaling

Maternal obesity has a profound impact on the hormonal and metabolic environment to which the fetus is exposed. Elevated maternal levels of insulin, glucose, leptin, and lipids cross the placenta, altering the development of fetal endocrine organs such as the pancreas, liver, hypothalamus, and adipose tissue [18].

One of the key alterations involves fetal hyperinsulinemia, which results from maternal hyperglycemia and excessive nutrient supply. This condition promotes islet cell hyperplasia in the fetal pancreas and increases insulin secretion, which not only contributes to macrosomia but also establishes a phenotype of insulin resistance that may persist postnatally [33].

It is suggested that adipokines play one of the key roles in fetal programming [34]. These molecules participate in the regulation of appetite and energy balance, as well as in processes such as angiogenesis, inflammation, immune function, blood pressure control, insulin sensitivity, glucose regulation, nutrient transport, and lipid metabolism. Their synthesis is altered in obesity, type 2 diabetes and metabolic syndrome [35].

Among the adipokines, leptin is considered to play the most important role. The concentrations and adipose tissue mRNA expression of leptin are strongly correlated with BMI and white adipose tissue mass [36]. Moreover, elevated fetal leptin levels may indicate increased adiposity but also contribute to leptin resistance in the developing hypothalamus. Since leptin plays a pivotal role in the regulation of energy homeostasis and appetite, such changes may disrupt neural circuits that control feeding behavior and energy expenditure [37].

In pregnancy, circulating leptin concentrations rise to two–three times the levels observed in non-pregnant women, with the maximum levels at 28th week and returning to pre-pregnancy levels after birth. The placenta is a major source of the maternal circulating leptin, and its levels are positively associated with the fat mass during gestation [38].

Although circulating leptin concentrations generally reflect adipose tissue mass, studies have demonstrated that obese individuals exhibit elevated leptin levels without corresponding anorexigenic effects. This phenomenon suggests the presence of leptin resistance in obesity [39]. Similarly, pregnancy has also been characterized as a physiological state of leptin resistance [40].

Dysregulated adiponectin synthesis may also play a significant role in these processes. Adiponectin, an adipocyte-derived hormone with insulin-sensitizing, anti-inflammatory, and vasculoprotective properties, plays an important role in regulating maternal-fetal metabolic homeostasis. Adiponectin is considered as an adipokine with insulin-sensitizing, anti-inflammatory, and anti-atherogenic properties. In non-complicated pregnancy, adiponectin contributes to glucose transport, lipid metabolism, and placental vascular function, thereby supporting appropriate fetal growth. It enhances glucose uptake in skeletal muscle while simultaneously suppressing hepatic glucose production [41]. A reverse association between the adiponectinemia and obesity (particularly the abdominal), as well as a strong positive correlation between the adiponectin levels and the insulin sensitivity, has been described [42]. Experimental research suggests that adiponectin may play a protective role in mitigating metabolic disorders associated with obesity [43]. It has been also noticed that during gestation the maternal adiponectin secretion gradually decreases. Low maternal adiponectin has been linked to increased placental expression of nutrient transporters, enhanced fetal adiposity, and a higher risk of macrosomia [44]. Diminished adiponectin signaling may disrupt placental development, promote inflammation, and modify fetal metabolic programming, predisposing offspring to obesity, insulin resistance, and cardiometabolic disease in adulthood [43].

Adiponectin contributes to the reduction of ectopic lipid accumulation by promoting fatty acid oxidation and suppressing lipolytic activity within adipose tissue. In rodent models exhibiting insulin resistance, intravenous delivery of recombinant adiponectin has been shown to restore normal insulin sensitivity [45]. Furthermore, excessive maternal lipid transfer to the fetus can lead to hepatic lipid accumulation and altered expression of genes involved in lipid metabolism, thereby predisposing offspring to non-alcoholic fatty liver disease (NAFLD) and dyslipidemia later in life [46].

### 2.4. Inflammatory Responses

Low-grade systemic inflammation, characteristic of obesity, is another critical mechanism influencing fetal development. Obese pregnant women frequently exhibit elevated circulating levels of C-reactive protein (CRP) and pro-inflammatory cytokines, including tumor necrosis factor α (TNF-α), interleukin-1 β (IL-1β), and interleukin-6 (IL-6). These inflammatory mediators can either cross the placenta or alter its barrier function, thereby inducing a pro-inflammatory state within the fetal compartment.

Fetal exposure to inflammation during critical windows of development can interfere with neurodevelopment, immune system maturation, and metabolic programming. For instance, IL-6 has been implicated in the dysregulation of hypothalamic development, particularly in areas involved in energy balance regulation [47].

In animal models, maternal inflammation during pregnancy induces microglial activation and increased blood-brain barrier permeability in the fetus, potentially linking maternal diet-induced inflammation with neurodevelopmental disorders such as autism spectrum disorder (ASD) and attention-deficit/hyperactivity disorder (ADHD). Furthermore, inflammation may impair the development of insulin-sensitive tissues and adipocytes, contributing to a pro-inflammatory phenotype in offspring, with increased susceptibility to obesity, metabolic syndrome, and cardiovascular disorders [48].

Importantly, the combination of inflammatory signaling with altered nutrient availability and hormonal disturbances may act synergistically, amplifying the impact of each individual pathway on fetal programming [49].

## 3. Obesity and Insulin Secretion, Insulin Resistance, and Diabetes

Pregnancy is correlated with gradually increasing insulin resistance. Throughout pregnancy, physiological processes during each trimester enhance food intake, promoting fat accumulation and energy storage to meet the elevated metabolic demands of gestation and lactation. This effect is particularly observed in obese patients with excessive food intake [50].

### 3.1. The Role of Adipose Tissue

Evidence from both human and animal studies has identified two principal factors influencing metabolic health and insulin sensitivity. One key determinant is the capacity of subcutaneous white adipose tissue (sWAT) to effectively store excess lipids, thereby preventing their deposition in ectopic depots such as the liver, skeletal muscle, and visceral adipose tissue (VAT). On the other hand, the capacity of subcutaneous white adipose tissue to recruit and differentiate new adipocytes, through activation of adipogenesis and enhancement of lipid storage capacity, has been shown to lower the risk of metabolic disorders, including type 2 diabetes mellitus (T2DM), in individuals who are overweight [51]. In obesity, WAT expansion occurs via two mechanisms: hyperplasia, characterized by an increase in adipocyte number, and hypertrophy, defined by enlargement of existing adipocytes. The latter process is more closely associated with insulin resistance and chronic inflammation [51].

Obesity-related alterations in WAT generate intrinsic molecular signals that trigger localized inflammation. This inflammatory response can subsequently extend to the systemic level, contributing to the development of insulin resistance and T2DM, and also influencing the occurrence of similar disorders in fetuses [52,53,54]. Maternal obesity has been shown to promote the development of a chronic, low-grade inflammatory state in the offspring’s white adipose tissue, which is frequently associated with insulin resistance [55,56,57].

### 3.2. The Role of Pancreatic β Cells

Growing evidence indicates that epigenetic alterations affecting key metabolic genes involved in these processes in offspring play a pivotal role in the developmental programming of profound disturbances in systemic glucose homeostasis and type 2 diabetes mellitus (T2DM) in adulthood [58].

Type 2 diabetes mellitus (T2DM) only develops when insulin secretion is insufficient to compensate for insulin resistance. Several mechanisms influence this process, including reduced β-cell mass and altered expression of molecular regulators, both of which are shaped by the adverse intrauterine environment. Fetal exposure to excessive maternal food intake and obesity may lead to disturbances in the developmental program and serious dysfunctions of the fetal pancreatic β cells and adipose tissue. The β-cell lose their mass and decreased vascular density of islet and mitochondrial dysfunction modify their function.

Pancreatic islets from offspring of mothers with obesity or high-nutrient diets exhibit lower insulin content, diminished *Pdx1* expression in adult islets, and structural remodeling, including an increased presence of α -cells in the central regions of the islets [59,60].

Several transcription factors are crucial for the proper development of both the endocrine and exocrine components of the pancreas. The earliest stages of β-cell formation are highly dependent on the activity of neurogenin 3 (Ngn3), a key basic helix–loop–helix transcription factor. Another critical regulator, the homeobox-containing transcription factor Pdx1 (also referred to as IDX1, IPF1, STF1, XlhBox8, GSF, or IUF), performs dual functions—it is essential for the early morphogenesis of the endocrine and exocrine pancreas and later for the differentiation and maturation of β-cells. Experimental studies have demonstrated that targeted homozygous deletion of Pdx1 results in pancreatic agenesis in mice, while homozygous mutations in humans produce a comparable phenotype [61].

Significantly, in animal models, maternal obesity before and during gestation and lactation results in altered development of the pancreas associated with insulin resistance in the germline of the fetus. The mechanisms by which epigenetic modifications established during early development may extend beyond the first generation remain incompletely understood. Genuine transgenerational epigenetic inheritance is considered to occur only when phenotypic features persist in the second and subsequent generations, as these are transmitted through the germline, which is not directly affected by the original maternal nutritional exposure [62,63].

Gestational diabetes mellitus (GDM), which frequently occurs in obese pregnant women, also increases the risk of diabetes in their offspring. The offspring of mothers with GDM have increased birth weight, as well as a marker such as glycated hemoglobin (HbA1C). Transcription factors in pancreatic islet cells are highly sensitive to suboptimal intrauterine environmental conditions. It has been observed that maternal hyperglycemia is a major trigger of epigenetic modifications, increasing the risk of glucose intolerance in the offspring. It is suggested that maternal hyperglycemia during pregnancy adversely influences the methylation of genes involved in pancreatic endocrine function and elevates the offspring’s risk of developing diabetes [64]. Metabolic disturbances associated with GDM promote the accumulation of reactive oxygen species (ROS) resulting from antioxidant deficiency not only in the maternal but also in the fetal organism. This oxidative imbalance leads to long-term disruption of energy homeostasis, increasing the risk of childhood obesity and the early-onset of type 2 diabetes mellitus in offspring [65].

Interestingly, epidemiological data in humans indicate that excessive nutritional intake in grandparents may elevate the susceptibility to diabetes and cardiovascular disorders in subsequent generations. Moreover, an increased prevalence of obesity and associated metabolic abnormalities has been documented among offspring whose parents maintained normal body weight but whose grandparents were obese [66].

It has been also noticed that a high-nutrient intrauterine environment in obese patients can lead to fetal macrosomia. Experimental and clinical studies have demonstrated that macrosomia is an independent risk factor for both types of diabetes in adulthood [67].

## 4. Obesity as Predisposition to Obesity and Adipose Dysfunction in Offspring

It has been confirmed in animal studies that maternal obesity during pregnancy and lactation, gestational diabetes mellitus, and accelerated postnatal growth collectively predispose offspring to the development of obesity [68,69]. Meta-analytic studies have also identified birth weight as an important predictor of future obesity and T2DM. The association between birth weight, a marker of fetal nutrition exposure, and the likelihood of developing obesity in future life has been described as U-shaped. This pattern suggests that both low and high birth weights, corresponding to reduced or excessive fetal adiposity, are associated with a similar increased risk of obesity and metabolic disorders later in life [15].

The regulation of appetite and satiety begins to develop during pregnancy in precocial species, preparing the organism for postnatal nutritional adaptation. In humans, neurons responsible for appetite and satiety control emerge in the fetal hypothalamus during the first trimester; however, the establishment of functional neuronal circuits occurs primarily in the third trimester of pregnancy [70,71].

Offspring of obese mothers exhibiting dysfunctional white adipose tissue, characterized by hypertrophic adipocytes, chronic inflammation, and insulin resistance, demonstrate impaired WAT plasticity and abnormal expansion of adipocyte progenitor cells within subcutaneous WAT [72]. These alterations may result from exposure to a maternal obesogenic environment and the premature depletion of resident adipocyte progenitors during development, thereby promoting adipocyte hypertrophy rather than hyperplasia as the primary mechanism for energy storage later in life [73]. Furthermore, Godfrey et al. demonstrated a strong correlation between DNA methylation of the retinoid X receptor alpha (RXRα) promoter region in umbilical cord samples from neonates born to mothers with low carbohydrate intake throughout gestation and the degree of obesity observed at 6–9 years of age [74].

Exposure to maternal obesity during crucial stages of development affects the expression and activity of key adipogenic transcription factors, thereby limiting the adaptive expandability of WAT in later life [75,76].

It has been suggested in animal models that the regulation of *Zfp423* gene expression and function plays a crucial role. Gupta et al. hypothesized that *Zfp423* gene expression defines committed preadipocytes, and its expression persists throughout adipocyte differentiation. In animal studies, inactivation of *Zfp423* during WAT development results in inhibition of differentiation, specifically within sWAT [77,78].

Fetal macrosomia observed in pregnancies complicated by maternal obesity is thought to result, at least in part, from enhanced placental nutrient transfer, a process influenced by adiponectin concentrations. [79,80]. Circulating adiponectin levels are consistently reduced in obese individuals and remain suppressed throughout gestation. Lower maternal adiponectin during pregnancy has been linked to placental insulin resistance and impaired placental function, characterized by augmented nutrient transport and increased fetal growth [81,82].

Another well-established epigenetic pathway implicated in the elevated obesity risk among offspring of obese mothers involves the altered expression and activity of peroxisome proliferator-activated receptor γ (PPARγ). This effect arises from epigenetic regulation of the PPARγ gene in white adipose tissue, mediated through DNA methylation and histone modifications in the promoter region [78]. In obese patients, an increased ratio of visceral adipose tissue (VAT) to subcutaneous adipose tissue (SAT) is observed. It has been shown that PPARγ2 (peroxisome proliferator activated receptor—γ2), a key transcription factor in the activation of adipocyte metabolism, modulates gene expression in adipocytes [83]. Two isoforms of protein: PPARγ1 and PPARγ2 can be produced from the PPAR γ gene. An important target of PPARγ2 is adiponectin, which improves insulin sensitivity and regulates adipose tissue deposition [84,85]. For example, in mice with overabundant VAT accumulation, overexpression of adiponectin results in its redistributed from VAT to sWAT, which improves metabolic parameters [86].

Evidence indicates that adiponectin plays a crucial role in the link between SAT PPARγ2 activation and the reduction of metabolic complications. Adiponectin expression and secretion are higher in sWAT than in VAT [87]. Activated sWAT PPARγ2 prevents hypertrophy of adipocytes by increasing the number of small adipocytes, which boosts adiponectin concentration [88]. It is important to note the presence of two adiponectin receptors, AdipoR1 and AdipoR2, which play key physiological roles in maintaining insulin sensitivity and regulating glucose metabolism in vivo. Studies in insulin-resistant and obese mice have demonstrated decreased expression of these receptors in adipose tissue, a change that is associated with diminished adiponectin-mediated insulin-sensitizing effects [89].

## 5. Obesity and Hypertension and Cardiovascular Diseases

The association between obesity and increased propensity for cardiovascular disease is well known [90]. Moreover, unbalanced maternal nutrition may influence the fetus, resulting in hypertension in future life.

To date, multiple key mechanisms have been implicated in the developmental origins of hypertension in the offspring of obese patients, including oxidative stress, nitric oxide (NO) insufficiency, abnormal activation of the renin-angiotensin system (RAS), fetal endocrine and metabolic dysregulation, disturbances in nutrient-sensing pathways, epigenetic modifications, and gut microbiota imbalance, among others [91,92].

### 5.1. Oxidative Stress and RAS Dysregulation

Oxidative stress, defined as an imbalance between reactive oxygen species (ROS) and antioxidant defenses, can emerge in utero in response to maternal obesity, diabetes, undernutrition, and placental dysfunction, contributing to impaired endothelial nitric oxide bioavailability and abnormal vascular reactivity in the fetus. During fetal development, elevated ROS levels have been shown to disrupt vascular and renal development, including reduced nephron endowment. This exposure to unfavorable intrauterine environments often results in an overproduction of ROS that exceeds the capacity of antioxidant defense systems [93]. This imbalance contributes to fetal programming events that predispose offspring to oxidative stress-mediated hypertension in adulthood [93,94].

In parallel, RAS dysregulation has been suggested as an important hormonal mechanism linking early-life adversity to long-term blood pressure elevation. Activation of the classic RAS elicits vasoconstriction, oxidative stress, and inflammation, resulting in hypertension. Maternal metabolic stress has been shown to upregulate fetal renin, angiotensin-converting enzyme (ACE), angiotensinogen, and angiotensin II type 1 receptor (AT1R) expression. These changes, which are maintained postnatally and contribute to increased vascular tone and sodium retention [95]. Importantly, oxidative stress and RAS activation potentiate one another: angiotensin II stimulates ROS production via NADPH oxidase, while oxidative stress further enhances RAS signalling, creating a feed-forward loop that amplifies the programmed hypertensive phenotype [96]. Collectively, these findings highlight how adverse intrauterine environments can induce molecular and physiological alterations that increase of hypertension later in life. Similarly, reduced nitric oxide (NO) bioavailability during pregnancy has been identified as a factor promoting programmed hypertension in later life.

### 5.2. Fetal Endocrine and Metabolic Dysregulation

During pregnancy, nutrient-sensing pathways play a vital role in adjusting fetal metabolism according to maternal nutritional status [97]. These signaling cascades monitor both intracellular and extracellular nutrient levels, such as lipid concentrations, and integrate them through systemic hormonal regulation [98]. Consequently, disturbances in maternal nutrition can disrupt these nutrient-sensing networks, exerting a profound influence on the developmental origins of hypertension [99].

In animal studies, maternal consumption of a high-fat diet (HFD) has been shown to induce hypertension in offspring, accompanied by hypomethylation of the leptin gene promoter and elevated leptin expression in adipose tissue of exposed rat progeny [100]. Likewise, other investigations have demonstrated that maternal HFD exposure may enhance sympathetic excitatory activity through increased leptin receptor expression, contributing to hypertension in adult offspring of rabbits [101]. It has been emphasized that a maternal high-fat diet during pregnancy is a key determinant of the offspring’s vasodilatory response to acetylcholine. This alteration disrupts fundamental endothelial function and predisposes the offspring to hypertension and major cardiovascular diseases [102].

The importance of cortisol is also emphasized in the pathogenesis of offspring hypertension. Numerous studies have reported positive correlations between cortisol levels, obesity and metabolic syndrome [103,104]. It is suggested that when 11β-HSD-2 (type 2 isoform of 11β-hydroxysteroid dehydrogenase) works properly, it serves as a crucial placental barrier limiting the transfer of cortisol from maternal to fetal circulation. This enzyme catalyzes the conversion of biologically active cortisol into its inactive metabolite, cortisone. Reduced placental 11β-HSD2 activity results in increased fetal exposure to maternal glucocorticoids, thereby altering the function of the hypothalamic-pituitary-adrenal (HPA) axis and predisposing the offspring to the development of hypertension and metabolic disorders later in later [105]. Guénard et al. suggested a positive correlation between elevated maternal body mass index (BMI), increased serum levels of glucose, total cholesterol, and low-density lipoproteins (LDL) and the development of cardiometabolic syndrome in the offspring [106].

It has been noticed that hyperglycemia, frequently observed in obese patients during early pregnancy, can lead to placental dysfunction, resulting in inflammatory responses that cause reduced blood flow and vascular constriction [10]. A similar effect may be elicited by glucocorticoids released in response to intrauterine stress. It is suggested that excess glucocorticoids throughout the crucial period of organogenesis induce vascular dysfunction, persistent alterations in hormonal secretion, and abnormal response to angiotensin II. These changes are responsible for an increased risk of hypertension and a greater predisposition to cardiovascular diseases in the offspring [8].

### 5.3. Gut Microbiota Dysbiosis

The gastrointestinal tract reside various microbes that coexist symbiotically with the host, exerting significant influence on health and disease [107]. Throughout pregnancy and lactation, maternal gut microbiota and their metabolites are transferred to the offspring, highlighting the pivotal role of maternal factors in the early development of the neonatal gut microbiome [108]. The gut microbiome is increasingly recognized as a critical mediator in the developmental programming of offspring health, particularly in the context of maternal obesity. Maternal obesity is associated with significant alterations in maternal gut microbial composition. These microbial changes can influence maternal metabolism, nutrient availability, and systemic inflammation, which collectively shape the intrauterine environment and fetal development. Altered colonization patterns in offspring may predispose to early-life metabolic dysfunction, including increased adiposity, insulin resistance, and dysregulated lipid metabolism. Maternal exposure to a high-fat diet has been shown to elevate blood pressure in the offspring, an effect associated with shifts in microbiota-derived metabolic profiles [109]. Several microbial metabolites, including short-chain fatty acids (SCFAs), trimethylamine (TMA), and its hepatic oxidation product trimethylamine-N-oxide (TMAO), play key roles in the regulation of vascular tone and blood pressure homeostasis.

Maternal consumption of a high-fat diet has been shown to diminish α-diversity within the offspring’s gut microbiota [110]. A reduction in α-diversity is one of the most consistent indicators of microbial dysbiosis and has been implicated in the pathogenesis of numerous diseases [111]. Furthermore, maternal high-fat diet exposure has been linked to the development of hypertension in progeny, accompanied by an elevated Firmicutes/Bacteroidetes (F/B) ratio. This pattern aligns with observations from experimental hypertension models, in which an increased F/B ratio has emerged as a characteristic microbial signature of hypertensive states [112].

Thus, the maternal gut microbiome represents a critical, modifiable interface between maternal metabolic status and fetal development, providing novel insights into mechanisms underlying the intergenerational transmission of obesity and hypertension and metabolic disease. Interventions targeting the maternal or neonatal gut microbiome, through diet, prebiotics, probiotics, or fecal microbiota transplantation, are being explored as potential strategies to mitigate adverse programming effects and improve long-term offspring metabolic health.

Animal studies have demonstrated that maternal overnutrition can induce structural and functional alterations within the cardiovascular system of the offspring. These alterations include endothelial dysfunction, enhanced sympathetic activity, myocardial connective tissue accumulation, and cardiac fibrosis-changes that are likely to compromise myocardial contractility and increase the susceptibility to cardiac dysfunction in later life [113,114].

Interestingly, it has been noticed that the mortality rate caused by coronary artery disease was significantly increased in patients with lower birth weight and lower weight at one year after labour, compared to individuals of appropriate or increased birth weight and this association was independent of other factors [115].

The relationship between maternal obesity during pregnancy and the subsequent risk of cardiovascular disease in offspring has not yet been examined in studies with adequately large cohorts, sufficient statistical power to assess dose-response associations, or follow-up periods long enough to elucidate potential causal mechanisms. Furthermore, maternal adiposity reflects not only genetic predisposition but may also affect the early-life environmental exposures that contribute to disease risk in offspring.

Thus, further research is required to elucidate the underlying mechanisms contributing to the development of hypertension and cardiovascular diseases in offspring of obese mothers.

## 6. Obesity as Risk Factor for Congenital Heart Defects in Offspring

Maternal obesity is recognized as significant risk factor to the development of congenital heart disease (CHD) in offspring [114].

Cardiac development occurs predominantly within the first trimester of pregnancy and is largely completed by approximately the sixth week of gestation; therefore, maternal metabolic and physiological conditions during early pregnancy are particularly critical for proper fetal heart development. Over the past decade, substantial progress has been made in elucidating the genetic determinants of congenital heart disease (CHD). More recently, large-scale epidemiological studies have identified correlations between maternal cardiometabolic conditions, such as obesity, and an elevated likelihood of CHD in offspring [116,117]. The significant phenotypic overlap between obesity, diabetes, and cardiometabolic disturbances remains challenging to explain, and it is still unclear which of these maternal factors influences directly on fetal risk during early pregnancy [115]. Multiple recent meta-analyses have consistently demonstrated a positive correlation between maternal overweight or obesity and an elevated risk of CHD in offspring. Maternal obesity has been correlated with a wide range of different cardiac defects, including patent ductus arteriosus, septal defects, aortic arch defects, conotruncal defects, left ventricular outflow tract obstruction defects, and right ventricular outflow tract defects [118,119,120].

Because noncritical forms of congenital heart disease may remain asymptomatic at birth and be diagnosed only later in life, existing studies are likely to underestimate the true incidence of CHD. Furthermore, much of the available evidence is derived from case-control analyses, which provide risk estimates that can be less reliable than those obtained from prospective, population-based cohort studies.

The risk of congenital defects is also associated with the degree of obesity. Persson et al. noticed that the likelihood for congenital malformations, including CHD, rises progressively as BMI increases from overweight to severe obesity [121].

The exact mechanisms through which maternal obesity affects key stages of cardiac morphogenesis remain unclear and are thought to involve multiple interacting factors. It has been suggested that abnormal glucose metabolism do not fully explain the risk of CHD in fetuses of obese mothers. Apart from carbohydrate metabolism disorders, a wide range of metabolic disturbances are observed in obese patients.

Maternal obesity is correlated with hyperinsulinemia, insulin resistance, dyslipidemia, and increased low-density lipoprotein susceptibility to oxidation, but the link between these abnormalities and the occurrence of CHD is not yet fully understood. Oxidative stress, impaired antioxidant defense, and altered cell signaling have been proposed as key mediators. However, maintaining optimal maternal glycemic control and body weight during pregnancy plays a crucial role in reducing the risk of CHD in the developing fetus [122].

It has also been suggested that the observed increase in CHD prevalence among offspring of obese mothers may, in part, reflect reduced prenatal detection rates during ultrasound scan. This diagnostic limitation arises because visualization of fetal cardiac structures is often suboptimal in pregnancies complicated by maternal obesity. Several studies have reported decreased ultrasound sensitivity for detecting cardiac anomalies in this population. Although data on pregnancy termination rates remain difficult to compare across studies, lower detection efficiency could plausibly result in fewer terminations following prenatal diagnosis, thereby increasing the proportion of CHD-affected pregnancies carried to term among obese women [123,124,125].

Adherence to a balanced diet in the year preceding conception has been linked to a reduced incidence of conotruncal and septal heart defects in offspring [126]. Furthermore, a maternal dietary pattern rich in one-carbon nutrients, characterized by high consumption of fish and seafood, has been correlated with a lower overall risk of congenital heart disease. Maternal malnutrition, particularly folate deficiency, has long been implicated in the etiology of CHD, and evidence suggests that women with obesity may exhibit a suboptimal response to folic acid supplementation intended for the primary prevention of congenital malformations [127].

Despite various lifestyle-based interventions targeting obese pregnant patients or a history of gestational diabetes mellitus, no consistent benefits have been observed in terms of gestational weight control or obstetric and perinatal outcomes [128]. Reduced adherence to dietary recommendations among women with obesity has been proposed as a contributing factor. Additionally, pre-pregnancy body mass index appears to be a stronger determinant of adverse pregnancy outcomes than gestational weight gain alone [129]. These findings confirm the importance of implementing preventive lifestyle measures before conception. Notably, emerging data indicate that specific genetic variants may modulate the efficacy of such interventions, suggesting the potential for more personalized strategies in the future [130].

Finally, maternal obesity represents a modifiable risk factor for CHD, with established, evidence-based strategies available to improve overall and maternal health outcomes. Modifiable lifestyle behaviors, including body weight management, physical activity, and nutritional patterns, represents key targets for preconception and antenatal interventions aimed at reducing the risk of CHD in offspring.

## 7. Potential Interventions

Considering recent scientific evidence highlighting the importance of the preconception period, particularly the influence of maternal body mass index before gestation and prenatal factors on the long-term health of the offspring, it appears crucial to ensure that women are properly prepared for pregnancy.

The aim is not to place full responsibility for the child’s metabolic health on the mother, but rather to emphasize that proper preparation for pregnancy, including maintaining a healthy weight, balanced nutrition, and regular physical activity, plays an important role in the future well-being of the child. Global nutrition education initiatives that support women throughout the preconception, pregnancy, postpartum, and early childhood periods may be particularly beneficial in this context. Preconception weight reduction through structured lifestyle interventions, such as individualized nutritional counseling, caloric optimization, and increased physical activity, has been shown to improve insulin sensitivity, reduce systemic inflammation, and lower the risk of adverse pregnancy outcomes. During gestation, targeted dietary modifications, including balanced macronutrient intake and limiting excessive gestational weight gain, may attenuate maladaptive intrauterine exposures that contribute to offspring metabolic dysfunction. Within such interventions, special attention should be directed toward pregnant women who were unable to manage overweight or obesity prior to conception. These women should be included in programs designed to promote healthy eating habits and encourage daily physical activity. Implementing such strategies can yield long-term benefits for both maternal and child health, extending from early life into adulthood.

Emerging strategies, such as modulation of the maternal gut microbiome through prebiotics, probiotics, or synbiotics, hold promise for altering inflammatory and metabolic pathways implicated in fetal programming. Probiotics and prebiotics have long been known for their benefits in human health. In animal studies it has been found that probiotic treatment with *Limosilactobacillus fermentum* or *Lactobacillus casei* during pregnancy and lactation prevents the development of hypertension in adult offspring exposed to maternal HFD.

The inherent reversibility of epigenetic alterations, combined with recent progress in epigenome-targeted technologies, offers promising perspectives for the development of novel therapeutic strategies. Modulation of the epigenetic machinery during early developmental periods represents a potential intervention to reduce the negative effects associated with maternal obesity.

A potential goal seems to be to focus on transient and reversible epigenetic modifications during the early stages of life, where pharmacotherapy or, in the future, genetic methods can be used to counteract the adverse effects of fetal programming induced by obesity. However, further research is required, and such interventions are not currently possible. At present, efforts should be primarily focused on investigating the relationships between maternal dietary quality, pre-pregnancy body weight, and the metabolic outcomes observed in their offspring.

## 8. Summary and Limitation

The prevalence of overweight and obesity is increasing worldwide among women of reproductive age, children, and in all other age groups. With the growing obesity epidemic, an increasing number of pregnancies are affected, leading to a heightened risk of metabolic disorders in the progeny. The consequences of maternal obesity extend well beyond birth, as the intrauterine environment during key developmental periods can induce long-lasting alterations in fetal physiology with implications for subsequent generations. Elucidating the mechanisms of fetal and offspring morbidity is essential for developing targeted interventions aimed at preventing or mitigating the adverse programming effects associated with maternal obesity.

The association between maternal obesity during pregnancy and the subsequent risk of diseases in offspring has not yet been evaluated in studies with sufficiently large cohorts, adequate statistical power to detect degree of obesity relationships, or follow-up durations long enough to clarify potential causal pathways. Moreover, maternal adiposity reflects not only genetic susceptibility but can also influence early-life environmental conditions that affect the risk of diseases in the offspring.

Despite the theoretical appeal of interventions (diet, exercise, metabolic control) during preconception or pregnancy, human trials demonstrating that such interventions can permanently reverse or attenuate fetal programming effects are limited. There is a need for prospective randomized controlled trials that follow children into childhood and beyond to assess long-term metabolic outcomes.

Another significant limitation is that the majority of studies on fetal programming focus on relatively homogeneous populations, often in high-income countries. There is insufficient data regarding from diverse socioeconomic, ethnic, and geographic groups, which limits generalizability of the findings. Without broader representation, it’s uncertain whether observed mechanisms and risks hold across different populations.

## Data Availability

No new data were created or analyzed in this study. Data sharing is not applicable to this article.
